# Generating 2.5D pathology for enhanced viewing and AI diagnosis

**DOI:** 10.1016/j.jpi.2025.100463

**Published:** 2025-07-18

**Authors:** Ekaterina Redekop, Mara Pleasure, Zichen Wang, Anthony Sisk, Yang Zong, Kimberly Flores, William Speier, Corey W. Arnold

**Affiliations:** a Department of Bioengineering, University of California, Los Angeles, USA; b Medical Informatics, University of California, Los Angeles, USA; c Department of Pathology, University of California, Los Angeles, USA; d Department of Radiological Sciences, University of California, Los Angeles, USA

**Keywords:** Digital pathology, 2.5D biopsy, Digital viewer, Deep learning, Prostate cancer, Vision transformer

## Abstract

Histological analysis of biopsy samples by pathologists can require the evaluation of complex three-dimensional (3D) tissue structures. This process involves studying the same tissue region across slides, which requires laborious zooming and panning for localization. Additionally, standard deep learning frameworks typically focus on cross-sections cut from biopsy specimens, limiting their ability to capture 3D tissue spatial information. We present a novel framework that constructs 2.5D biopsy cores via the extraction and co-alignment of serial tissue sections using a novel morphology-preserving alignment framework. These 2.5D cores can then be used for enhanced viewing by pathologists and as input to video transformer models that can capture depth-wide spatial dependencies. We used our framework to construct 2.5D cores for 10,210 prostate biopsies, 156 breast biopsies, and 1869 renal biopsies. To evaluate the utility of the cores for downstream tasks, we performed additional studies in prostate cancer by: (1) training a deep learning-based cancer grading model and (2) conducting a reader study with pathologists.

## Introduction

Tissue acquired for pathological analysis is inherently three-dimensional (3D), but routine clinical processing requires cutting and staining serial sections from tissue blocks. This procedure impedes the appreciation of important volumetric morphology, cellular architecture, and the spatial distribution of pathological structures. The number of cuts can vary between tissue types and labs, but up to 48 sections can be obtained from a single needle core biopsy.^1^, ^2^, ^3^ In specialties where volumetric information is important for diagnosis, such as prostate, diagnosis requires the time-consuming and mundane process of swapping slides on the microscope and panning/zooming to the same location across slides.

Recently, non-destructive 3D imaging technologies such as open-top light-sheet microscopy (OTLS) have emerged to better characterize the morphology in a tissue volume.^4^, ^5^, ^6^, ^7^ However, clinical translation of these technologies has been limited due to the complexity of manual evaluation, the absence of computational platforms to analyze complex 3D tissue arrays, and the fact that existing digital pathology applications employing 3D imaging typically operate at a maximum magnification of 10×, which is two times smaller than the resolution typically required for detailed cellular analysis. These limitations have led to the development of deep learning (DL)-based tools to process 3D pathology images and predict patient outcomes. MAMBA^8^ and its extension TriPath^9^ are two such tools that use attention-based multiple instance learning (ABMIL).^10^ They were trained in a weakly supervised manner to predict patient-level risk for prostate cancer (PCA) across two different imaging modalities: 50 simulated core needle biopsies from prostatectomy specimens imaged with OTLS and 45 prostatectomy specimens imaged with microcomputed tomography (microCT). A main limitation of the study is the relatively small size of the datasets, each with only 50 and 45 samples for OTLS and microCT, respectively, greatly restricting the generalizability and robustness of the model's predictions. Secondly, another limitation is MAMBA and TriPath lack a pathology-specific feature encoder, crucial for capturing the intricate details needed for high-quality data representation in digital pathology.

**Fig. 1:**
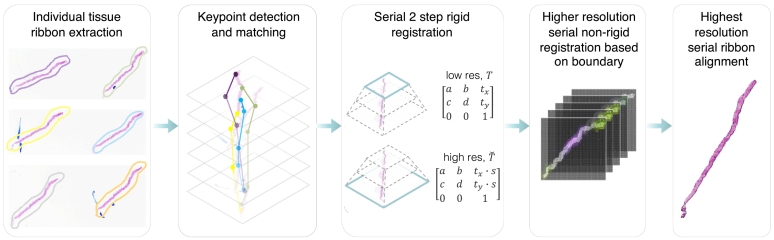
2.5D pathology generation workflow. The morphology-preserving tissue alignment framework consists of three main steps: individual tissue ribbon extraction, serial rigid registration, and high-resolution non-rigid registration based on the boundary.

**Fig. 2:**
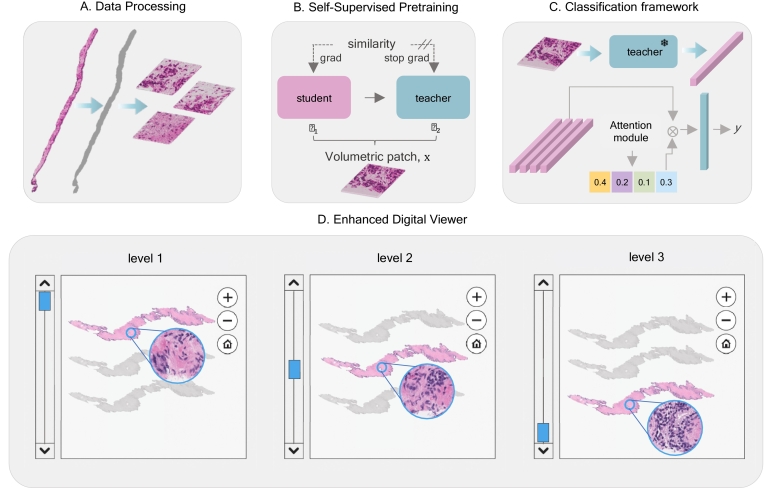
Enhanced viewing and AI diagnosis based on 2.5D pathology. (A) The framework first separates the core tissue from the background and then splits it into the 2.5D patches without overlap, removing patches containing less than 60% of tissue.) (B) Self-supervised pretraining framework based on DINO with TimeSformer backbone for spatiotemporal feature learning directly from a 2.5D patch.) (C) The 2.5D patches are processed with a pretrained feature encoder network. The resulting set of features is combined using a learnable attention module to produce a final patient-level prediction.) (D) Clinical validation using a digital slide viewer with the ability to scroll through consecutive and co-registered tissue sections of a 2.5D core.)

The transition to whole-slide imaging (WSI) presents opportunities for computational techniques that can fuse information across two-dimensional (2D) slides to better represent the 3D nature of tissue. In this work, we describe a novel morphologypreserving alignment framework to construct a 2.5D core from routinely scanned 2D biopsy WSIs (see Fig. 1) and evaluate its relative utility in DL diagnosis and in a reader study with pathologists (see Fig. 2). Pathology image alignment has two major difficulties: (1) aligning serial tissue sections while maintaining morphological integrity and (2) applying a complex registration process to gigapixel images. The VALIS framework^11^ was developed to address these challenges by performing automatic preprocessing, normalization, tissue detection, feature extraction, and matching for automatic tissue alignment. The SuperGlue Graph Neural Network keypoint matching framework^12^ was developed to improve traditional feature matching algorithms used in VALIS (RANSAC, Tukey's approach, and neighbor match filtering) by utilizing graph neural networks to solve a differentiable optimal transport problem. Previous work has focused on performing registration with features extracted directly from the ribbon rather than solely from the ribbon boundaries, which leads to the increased risk of altering the tissues morphology.^11^^,^^13^^,^^14^ Our approach builds upon VALIS and SuperGlue by using the ribbon's boundary to perform non-rigid registration to preserve nuclei and glandular morphology. The developed co-registration framework operates on WSIs with magnification up to 20× (0.5 μm/pixel), which is higher than data obtained with novel 3D technologies^9^ and therefore provides more tissue morphology details which are essential for cancer grade prediction.^15^

The DL-based diagnosis pipeline we used is based on ABMIL^10^ with a novel video transformer-based feature encoder (Fig. 2C). We implemented a modification of the widely used DINO contrastive learning framework,^16^ optimizing the feature encoder architecture to work with 2.5D biopsy cores, which we treat as a series of video frames (Fig. 2B). For our reader study, we evaluated a slide viewer that allows for rapid scrolling through 2.5D cores, which we compared to conventional microscope-based signout of glass slides (Fig. 2D).

## Materials and methods

### Data

We construct a 2.5D biopsy dataset by applying our alignment framework to 10,210 prostate biopsy cores, 156 breast cores, and 1869 renal cores coming from in-house data. Each core was serially sectioned to 6–16 ribbons and placed on three glass slides. Tissue was stained with hematoxylin and eosin (H&E) and scanned at 40× magnification (0.25 μm/pixel). Digitized WSIs were downsampled to 20× magnification and transformed into 2.5D cores.

Prostate 2.5D cores were further processed for our DL diagnostic evaluation to classify ISUP Grade Group (GG). For each core, tissue masks were generated slice by slice by converting downsampled slides to the HSV color space and applying a threshold to the hue channel. Morphological closing was then used to refine these tissue masks by filling small gaps. Non-overlapping 2.5D patches of size 256×256×*Z* with more than 60% tissue were extracted from the slide, where *Z* - number of ribbons in the core (see Fig. 2A).

We randomly divided this dataset by patient into 70% for training, 10% for tuning (validation), and 20% for testing. We stratified the dataset by patient-level GG determined by the max GG in each patient's set of biopsy cores, resulting in 5384 cores for training, 772 for tuning (validation), and 1559 for testing. (Table I).

**Table I:** Number of cores for each GG.

Grade group	Train	Tune (Validation)	Test	Total
BN	3563	502	978	5043
GG 1	711	97	220	1028
GG 2	564	90	186	840
GG 3	272	49	89	410
GG 4/5	274	34	86	394

A subset of 25 cases from the test subset was selected for a reader study with pathologists. This subset included five cases from each GG group.

### Morphology preserving alignment

The alignment framework is built upon VALIS^11^ and SuperGlue Graph Neural Network keypoint matching.^12^ The framework consists of: (1) extracting individual tissue ribbons from each downsampled 2D WSI using morphological labeling, (2) aligning individual tissue ribbons to obtain initial components for rigid transformation, and (3) performing rigid and non-rigid registration of the corresponding high-resolution WSIs. Initial keypoints and descriptors are obtained using the scale-invariant feature transform key-point extractor.^17^ Using the set of keypoints and corresponding visual descriptors for each unregistered pair of images, the assignments are estimated by solving a differentiable optimal transport problem with costs predicted by a graph neural network.^12^ The estimated transformation matrix has only rotation, translation, and scaling components to eliminate tissue deterioration by non-rigid deformations. Due to the linear relationship across the WSI levels, an estimation of the translation vector at the original resolution can be calculated by multiplying the result with the corresponding downsampling factors. The scaling factors and the rotation angle are independent of the resolution and can be directly applied to the higher resolution levels. Rigid registration is performed sequentially, aligning each image in the stack to the reference image. The first image is the reference, then the second image is aligned to it, then the third is aligned to the registered version of the second image, and so forth. Only features present in both neighboring images are used to align each image to the next one in the stack. The rigidly aligned images are stacked together to create a non-rigid registration mask.

Subsequently, the bounding box of this mask is utilized to extract higher-resolution versions of the tissue from each slide at 20× magnification, which are used for non-rigid registration. To perform non-rigid registration, we find 2D displacement fields by optimizing metrics based on ribbon boundaries using the SimpleElastix method.^18^ Ribbon boundaries are obtained by finding ribbon masks from each rigidly aligned ribbon. After the displacement fields are found, the resultant transformation is applied to the original non-binary version of the image.

### Slide-level classification

Due to the gigapixel size of WSIs, patching is commonly used to train deep-learning models. However, patch-based annotations are time-consuming and expensive to obtain, instead slide-level labels which are more readily available can be used as a weak local label. In this scenario, multiple instance learning (MIL) can be used to represent each WSI as a ‘bag’ or set of instances.^19^ Attention-based MIL classifies the entire bag of instances instead of individual instances by using a trainable attention module to learn the relative importance of each instance for the final prediction.^10^ Localization is achieved by aggregating the learned attention values into a final attention map. The attention module operates on deep features extracted by a pretrained encoder.

### Video transformer

To pretrain the feature extractor for 2.5D cores, we used the DINO framework, in which a student network was trained to match the probability distribution of a siamese teacher network using contrastive loss.^16^ Whereas “global” (224 × 224 resolution, covering larger regions of the image) views of the image are passed through the teacher network, “local” (96 × 96 resolution, covering smaller regions) are passed through the student network, encouraging “local-to-global” correspondences learned through the contrastive objective. In the DINO framework, teacher and student networks share the same architecture, and the teacher is optimized using a momentum encoder. The framework was adapted for the 2.5D cores by substituting a regular visual transformer (ViT)^20^-based architecture for student and teacher networks with TimeSformer for spatiotemporal feature learning directly from a volumetric patch.^21^ The TimeSformer framework was built on top of standard transformer architecture to enable spatiotemporal feature learning by sequentially applying temporal and spatial attention. In this work, the temporal component is adapted to function as a depth component within the 2.5D patch. Following the ViT setting, each 2D section from the 2.5D patch is decomposed into *N* non-overlapping patches of size P×P, which are flattened into vectors $x_{\left(p,t\right)}\in R^{3P^{2}}$, where p=1,…,N denoting spatial location and t=1,…,F-index over z-stack of 2D slices. Each patch $x_{\left(p,t\right)}$ is mapped into an embedding vector using learnable matric $E\in R^{D\times 3P^{2}}$ and combined with a positional embedding $e_{p,t}^{\mathrm{pos}}\in R^{D}$ to encode spatiotemporal position:

(1) $$z_{p,t}^{0}=Ex_{p,t}+e_{p,t}^{\mathrm{pos}}$$

The resulting vector represents the input to the Transformer, which consists of *L* encoding blocks. For each block l, the representation $z_{p,t}^{l-1}$ from the preceding block is used to compute a query (*q*), key (*k*), and value (*v*) vector for each patch:

(2) $$q_{\left(p,t\right)}^{\left(l,a\right)}=W_{Q}^{\left(l,a\right)}\mathrm{LN}\left(z_{p,t}^{\left(l-1\right)}\right)\in R^{D_{h}}$$

(3) $$k_{\left(p,t\right)}^{\left(l,a\right)}=W_{K}^{\left(l,a\right)}\mathrm{LN}\left(z_{p,t}^{\left(l-1\right)}\right)\in R^{D_{h}}$$

(4) $$v_{\left(p,t\right)}^{\left(l,a\right)}=W_{V}^{\left(l,a\right)}\mathrm{LN}\left(z_{p,t}^{\left(l-1\right)}\right)\in R^{D_{h}}$$

where *LN* represents layer normalization,^16^ $a\in \left\{1,\ldots ,A\right\}$ is the index over attention head in a transformer block. For divided attention, temporal attention is computed first within each block *l*:

(5) $$\alpha_{\left(l,a\right)\left(p,t\right)}^{\mathrm{time}}=\mathrm{SM}\left(\frac{q_{\left(p,t\right)}^{{\left(l,a\right)}^{T}}}{\sqrt{D_{h}}}\cdot {\left\{k_{\left(00\right)}^{\left(l,a\right)},k_{\left(p,t^{\prime }\right)}^{\left(l,a\right)}\right\}}_{t^{\prime }=1,\ldots ,F}\right)$$

where *SM* - softmax activation function. The encoding $z_{p,t}^{\left(l\right)\mathrm{time}}$ is obtained by computing the weighted sum of value vectors using self-attention coefficients from each attention head. New key/query/value vectors are calculated from $z_{p,t}^{\left(l\right)\mathrm{time}}$ and it is then passed for spatial attention computation:

(6) $$\alpha_{\left(l,a\right)\left(p,t\right)}^{\mathrm{space}}=\mathrm{SM}\left(\frac{q_{\left(p,t\right)}^{{\left(l,a\right)}^{T}}}{\sqrt{D_{h}}}\cdot {\left\{k_{\left(00\right)}^{\left(l,a\right)},k_{\left(p^{\prime },t\right)}^{\left(l,a\right)}\right\}}_{p^{\prime }=1,\ldots ,N}\right)$$

The resulting vector $z_{p,t}^{\left(l\right)\mathrm{space}}$ is passed to the MLP to compute the final encoding $z_{p,t}^{l}$ of the patch for the block l.

### Model comparison

The proposed DINO pretraining, utilizing the 2.5D core, was compared to a baseline pretraining approach that used 2D image patches derived from slicing the 2.5D core along the z-axis. The baseline approach employed the default ViT backbone for pretraining. After pretraining, a frozen backbone was used to extract patch-level feature vectors, which were aggregated into a core-level vector through ABMIL.

Additionally, following the MAMBA and TriPath frameworks, we used a ResNet50-based feature extractor pretrained on video clips (Kinetics-400) followed by ABMIL for comparison.

### Clinical validation

We conducted a reader study with two experienced genitourinary pathologists (P1 and P2) for prostate GG grading based on 2.5D cores. We randomly sampled a cohort of 25 patients from our test subset with equal distribution among the five GG. In total, we organized two rounds of reader studies. In the initial round, both pathologists performed a conventional microscope-based examination of serial tissue sections, completing the spreadsheet as they do in their routine practice. The spreadsheet included the final diagnosis, GG, estimated percentage of the tumor's core occupied, estimated tumor size in mm, and the presence of cribriform, intraductal carcinoma, and perineural invasion. The pathologist selected the final diagnosis from one of the following categories: Benign, PCA, HGPIN (high-grade prostatic intraepithelial neoplasia), ASAP (atypical small acinar proliferation), PINATYP (high-grade PIN with ASAP), ASAP-HI (highly suspicious for carcinoma), AIP (atypical intraductal proliferation, suspicious for intraductal carcinoma), BFM (benign fibromuscular tissue, no prostatic tissue seen). Pathologists performed a similar assessment in the second round after a 3-month washout period using 2.5D core samples and a digital slide viewer with the ability to scroll through consecutive and co-registered tissue sections of a 2.5D core developed based on OpenSeadragon,^22^ a JavaScript library that allows building a viewer with advanced zooming support. The improved functionality allows scrolling through an unlimited number of co-registered tiles at up to 20× magnification.

### Evaluation metrics

To evaluate the morphology-preserving image alignment framework, registration error was calculated as the median distance (μm) between subsequent keypoints in the stack for each image. Core-based registration error was then calculated as the average of the registration errors collected for each image in the stack, weighted by the number of matched features per pair of images.

The performance of GG classification prediction using the ABMIL framework was evaluated using AUC, multi-class weighted Precision, Recall, and F1 score metrics. We utilized the McNemar statistical test to evaluate the significance of the difference in classification accuracy between the models.

We assessed the intra- and interpathologist agreement within each study type (microscope-based and digital) by computing the quadratic-weighted kappa metric.

We used the attention rollout method^23^ to visualize attentions for the TimeSformer backbone of the DINO contrastive learning framework. Assuming the attention weights determine the proportion of the incoming information that can propagate through the layers, these weights can be used to approximate how the information flows between network layers. If $A_{l}$ is a 2D attention weight matrix at layer *l*, $A_{l}\left[i,j\right]$ would represent the attention of token *i* at layer *l* to token *j* from layer *l* − 1. Attention to the input tokens from the 2.5D patch is computed by recursively multiplying the attention weights matrices, starting from the input layer up to layer l. Each token has two dimensions for divided space–time attention, i.e., *z*(*p,t*), where *p* is a spatial dimension and *t* is the depth dimension in the 2.5D patch. Each TimeSformer encoding block contains a time attention layer and a space attention layer. During the time attention block, each patch token only attends to patches at the same spatial locations, whereas during space attention, each patch only attends to the patches from the same frame. *T*[*p,j,q*] represents the attention of *z*(*p,j*) to *z*(*p,q*) from the previous layer during time attention layer, where *T* are the time attention weights. *S*[*i,j,p*] represents the space attention of *z*(*i,j*) to *z*(*p,j*) from the time attention layer, where *S* are space attention weights. Combining the space and time attention, each 2.5D patch token attends to all patches at every spatial location within the volume through a unique path. The combined space-time attention *W*:

(7) $$W\left[i,j,p,q\right]=S\left[i,j,p\right]*T\left[p,j,q\right]$$

## Results

### Morphology preserving alignment

Elastic registration reduced the registration error by 50% compared to rigid registration for prostate cancer biopsies, from 40.1 to 20.8 μm median distance. The proposed non-rigid registration improvement achieved a similar performance (20.1 μm) and reduced the risk of glandular shape deterioration, which was examined by manual evaluation. The registration error decreased from 186.9 to the 48.3 μm median distance by using the proposed morphology-preserving method, compared to rigid registration for breast biopsies cohort. For the renal cohort, the proposed morphology-preserving method reduced the registration error from 114.6 μm to a median distance of 23.7 μm, outperforming rigid registration. Examples of patches extracted from constructed 2.5D cores can be found in Supplementary fig. S1.

### DL-based cancer grading model

The results of the prostate cancer GG prediction are shown in Fig. 4A. Using 2.5D features in ABMIL significantly outperformed (McNemar‘s test, $\chi^{2}$ = 63.0, *p <* 0.001) other feature types and achieved the highest AUC of 0.958, F1 of 0.671, Precision of 0.661, and Recall of 0.695. It resulted in a 1% increase in AUC, a 6.5% increase in the recall, a 6.1% increase in precision, and a 5.8% increase in the F1 score compared to 2D features. ABMIL trained using features extracted with a Video ResNet model, utilized in the previous 3D pathology work, only obtained 0.7 AUC, 0.286 F1 score, 0.318 Precision, and 0.332 Recall, illustrating the importance of pathology-specific encoders. Fig. 4B illustrates the confusion matrix for the prediction of GG. The features extracted using 2.5D DINO demonstrate superior performance across all classes (as shown along the main diagonal) compared to those extracted using 2D DINO or Video ResNet models. The features extracted using 2.5D DINO achieve higher accuracy in detecting GG1 cases (66.3% vs. 62.7%) and a lower likelihood of misclassifying clinically insignificant GG1 as clinically significant (CS) GG2 (22.3% vs. 25.9%), which could influence treatment planning. The confusion matrix corresponding to Video Resnet indicates the model overfitting to the largest class (Benign).

Additionally, we binarized GG labels and predictions into CS (GG ≥ 2) vs not (GG *<* 2). We found that features extracted using 2.5D DINO outperform both 2D DINO and Video ResNet baselines in accuracy (0.808 vs. 0.795 vs. 0.625), F1 score (0.839 vs. 0.829 vs. 0.613), precision (0.813 vs. 0.797 vs. 0.740), and recall (0.866 vs. 0.862 vs. 0.520), as shown in Fig. 4C.

**Fig. 3:**
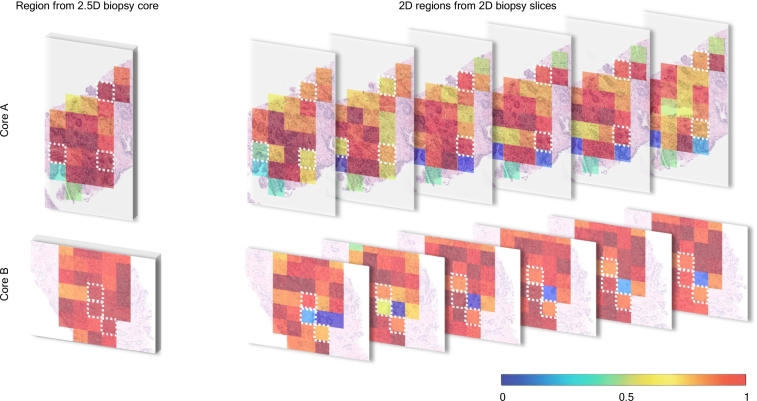
Visualization of discriminative regions from WSIs, overlaid with attention maps from the ABMIL module, highlighting two clinically significant cores (Core A and Core B). In these cases, ABMIL using features extracted with 2.5D DINO yielded a true-positive (TP) prediction, whereas ABMIL with 2D DINO features resulted in a false-negative (FN) prediction. White boxes indicate patches highlighted by pathologists. The overlaid jet colormap highlights model attention, where red indicates the highest attention and blue indicates the lowest. (For interpretation of the references to color in this figure legend, the reader is referred to the web version of this article.)

The results show that a 2.5D morphology-aware computational framework, which offers a natural approach to analyzing intrinsically 3D biological structures, has the potential to enhance the diagnostic accuracy of automatic solutions. We support our argument by visualizing two discriminative regions from WSIs overlaid with attention maps from two CS cores (see Fig. 3) correctly classified by our framework and misclassified by other models. Highlighted patches examined by pathologists were determined to be tumor patches containing volumetric information important to detecting clinical significance. With only a single 2D view of the tumor within these patches, there is a risk of either missing it or grading it inaccurately. In Fig. 3, we overlay the highlighted patches with attention maps from the TimeSformer backbone, highlighting important glandular variations within the volume essential for correct grading.

**Fig. 4:**
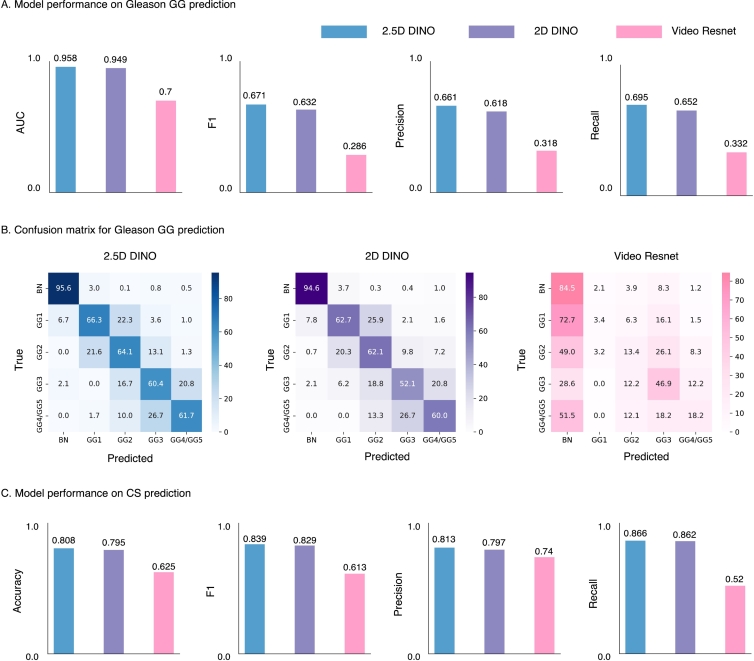
Prostate cancer ISUP GG classification results using ABMIL, leveraging features extracted by encoders pretrained on 2.5D pathology patches (2.5D DINO), 2D pathology patches (2D DINO), and Kinetics-400 video clips (Video ResNet). (a) AUC, F1, Precision and Recall for five class classification task. (b) Confusion matrix comparing actual and predicted labels for five-class classification task. (c) AUC, F1, Precision, and Recall for binary model performance (GG ≥ 2 vs. GG *<* 2).

**Fig. 5:**
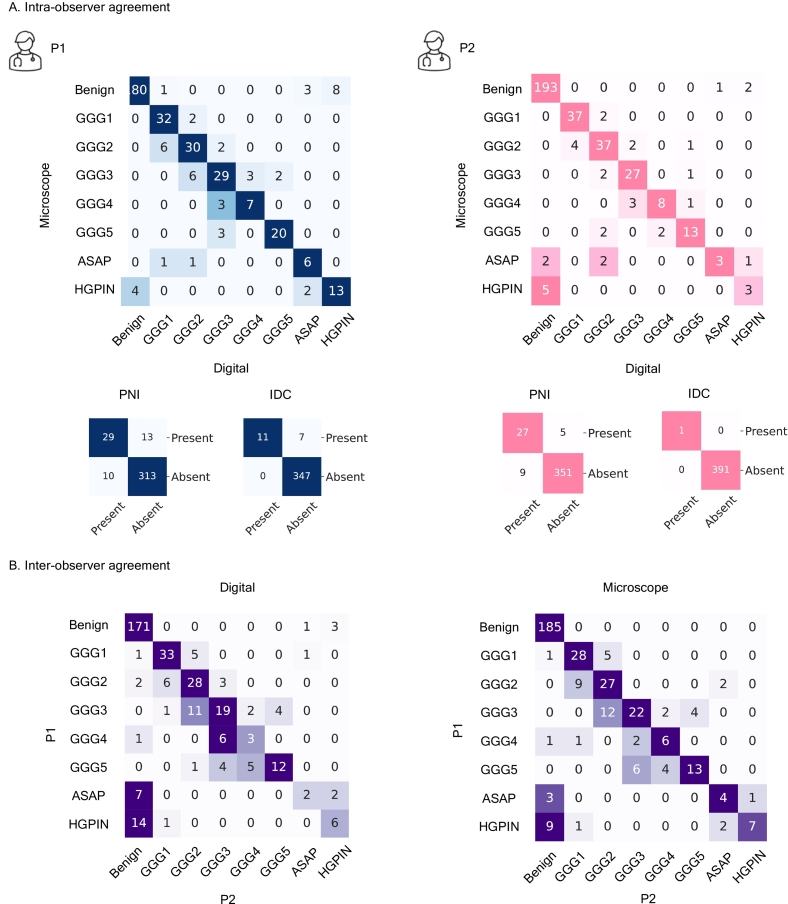
Results of the clinical validation of the 2.5D core using a digital pathology slide viewer with advanced functionality. (a) Confusion matrix illustrating intraobserver variability for two pathologists (microscope assessment vs. digital assessment using the 2.5D core). (b) Confusion matrix illustrating interobserver variability between two pathologists (microscope assessment and digital assessment using the 2.5D core. ASAP - atypical small acinar proliferation, HGPIN - high-grade prostatic intraepithelial neoplasia, PNI - perineural invasion, IDC - intraductal carcinoma.

### Clinical validation

We evaluate the intra- and interpathologists' agreement using quadratic Cohen kappa *κ*, comparing diagnostic differences between traditional microscope-based and digital assessments utilizing the 2.5D core. The interpathologists' agreement estimation resulted in *κ* = 0*.*7 for microscope-based examination and *κ* = 0*.*73 for digital examination utilizing a 2.5D core. The improved *κ* value for digital examination indicates better agreement among pathologists, reflecting increased consistency and reliability in the diagnostic process. The intrapathologist agreement estimation resulted in *κ* = 0*.*81 for P1 and *κ* = 0*.*87 for P2 comparing microscope-based to digital signout, indicating substantial agreement.

A confusion matrix was used to compare both sign-out results on a core-by-core basis (see Fig. 5A). Whereas most cases identified as benign through microscopic examination remained classified as benign after digital examination using a 2.5D core, a few cases were reclassified as HGPIN or ASAP by both pathologists. Only a few cases initially deemed clinically insignificant by microscopic examination were reclassified as CS by both pathologists (5.8% by P1 and 5.1% by P2). Conversely, a higher proportion of cases initially identified as CS through microscopic examination were subsequently classified as clinically insignificant after digital sign-out (15.7% by P1 and 9.3% by P2).

A confusion matrix was used to compare the diagnoses of P1 and P2 on a core-by-core basis (see Fig. 5B). 14.7% of cores graded as GG1 by P1 were graded as GG2 (CS) by P2 during the microscope-based examination and 12.5% during the digital examination utilizing the 2.5D core. 23.6% of cores graded as GG2 by P1 were graded as GG1 or benign by P2 during the microscope-based examination and 20.5% during the digital examination utilizing the 2.5D core. Additional analysis is presented in Supplementary fig. S2.

## Discussion

Distance metrics and visual inspection of our 2.5D alignment framework indicated the method was successful at aligning ribbons, a finding that was supported by our downstream analyses. The framework may then thus allow for much larger 2.5D cohorts compared to methods for generating 3D pathology. Whereas 2.5D images will not be as accurate as true 3D, the trade-off is acceptable given the ability to generate large volumes of data from retrospective cohorts at low cost. In addition to the downstream areas we explored, 2.5D pathology may be useful for other tasks, such as studying the tumor microenvironment.

Our classification results revealed a significant performance drop when using feature extractors pretrained on natural images, highlighting the importance of pathology-specific pretraining frameworks. Additionally, the proposed framework showed higher performance compared to traditional 2D approaches. This result supports our hypothesis that the proposed pretraining framework, which operates on 2.5D patches, provides additional value over conventional 2D-based pretraining approaches due to its ability to incorporate information across ribbons.

In our reader study, we observed that using digital examination with the 2.5D core resulted in a smaller diagnostic discrepancy between the readers compared to microscope-based examination. The reduced discrepancy promotes more consistent diagnoses across pathologists, which can help standardize patient diagnosis and subsequent management. Of note, a lower percentage of disagreement occurred between pathologists within CS and insignificant cases when using digital examination with the 2.5D core compared to the microscope. This result is particularly important given the very different interventions between the two categories (e.g., active surveillance versus definitive treatment).^24^ However, we note that a larger multi-center study with more pathologists would be needed to explore the advantages of digital interpretation of 2.5D cores more fully. Such a study could more robustly assess the variability in diagnostic accuracy and interobserver consistency across a diverse range of clinical expertise.

We acknowledge that the ultimate value of any pathology enhancement pipeline lies in its impact on downstream clinical or molecular prediction tasks. Whereas this study focuses on establishing a robust, scalable workflow for 2.5D pathology and evaluating its impact on diagnostic consistency and efficiency, we recognize that future work should explore how enhanced volumetric data can improve the prediction of patient outcomes and therapeutic response. By enabling consistent alignment and reducing sampling bias across sections, our method creates the foundation for training more reliable models for these applications.

## Conclusion

Our study suggests that 2.5D cores and the developed framework may offer benefits in a digital pathology environment, including the potential to improve diagnostic congruence and reduce review time, both of which can have a significant impact on histopathology practice. Future directions include the discovery of new 2.5D biomarkers that enhance different clinical and research applications.

## Declaration of competing interest

The authors declare that they have no known competing financial interests or personal relationships that could have appeared to influence the work reported in this paper.

## Appendix A Supplementary data

Supplementary video 1 Scrolling through a 2.5D prostate biopsy core using the digital viewer, illustrating the volumetric structure and spatial continuity across sections. Supplementary video 1

Supplementary material 2 Implementation details and additional analysis. Supplementary material 2
