# Supplementary material for: Generating 2.5D pathology for enhanced viewing and AI diagnosis
Source: J Pathol Inform. 2025 Jul 18;18:100463. doi: 10.1016/j.jpi.2025.100463 (PMC12355132; doi:10.1016/j.jpi.2025.100463)
Supplement: Supplementary material 2 — Implementation details and additional analysis. [file mmc2.docx]

**SUPPLEMENTARY MATERIALS**

# Implementation Details

Registration was executed entirely on CPU, while deep learning components were run on NVIDIA Tesla V100 GPUs (32 GB VRAM) using CUDA 11.7.

# TimeSformer Architecture

We utilized a modified version of the TimeSformer architecture based on Vision Transformer (ViT) backbones adapted for video-level input. The model consists of 12 transformer layers, each with 12 attention heads and an embedding dimension of 384. Input videos are divided into spatiotemporal tokens using a patch size of 16 × 16 and 8-frame clips.

The attention mechanism is based on a “divided space-time” strategy: temporal attention is applied separately from spatial attention within each block. Temporal attention is computed across frames at each spatial location, while spatial attention operates within each frame. The output class token aggregates information across all frames using a mean pooling strategy.

- **Input size:** 3 × 8 × 224 × 224 (channels × frames × height × width)
- **Patch size:** 16 × 16
- **Embedding dimension:** 384
- **Number of layers:** 12
- **Attention heads:** 12
- **Dropout rates:** 0.0 (embedding), 0.1 (stochastic depth)
- **Attention type:** divided space-time

We used pretrained ImageNet weights (ViT-B/16) for initialization. Positional encodings were resized to match the input resolution at inference. Code for the model is adapted from the publicly available TimeSformer implementation by Bertasius et al. [1].

FIG. S1. shows the progression of tissue slices as the user scrolls through the volumetric viewer interface. A jet colormap is overlaid to visualize model attention, with red indicating high attention and blue indicating low attention. Attention maps were extracted using the *TimeSformer rolled attention* tool [2]. In the context of prostate biopsies, these visualizations demonstrate that the model learns to attend to diagnostically relevant regions over volume, such as glandular architecture and luminal morphology. This supports the model’s ability to perform complex spatiotemporal reasoning, focusing on gland configuration and nuclear-stromal context.

# Timing Analysis of 2.5D Core Assessment Workflow

To evaluate the practical efficiency of a digital pathology slide viewer with advanced functionality, we conducted a systematic timing study comparing the duration of core assessment using an improved digital slide viewer with the ability to scroll through 2.5D core versus traditional manual serial section review. For each core, a timer was started at the beginning of the assessment process—defined as the moment the pathologist began reviewing the case (whether using the digital viewer or manual serial slides)—and stopped when the pathologist completed their evaluation. This includes the time spent scrolling through sections, identifying relevant features, and making diagnostic annotations or measurements. The timing was recorded separately for both pathologists across all evaluated cores. The results of this timing comparison are presented in FIG. S2. The plot shows the percentage reduction in core assessment time (i.e., speed-up) for the improved volumetric viewer designed for 2.5D core compared to manual review with paired data points for both pathologists. FIG. S2. demonstrates a consistent and statistically significant reduction in time to diagnosis when using the viewer with a 2.5D core. On average, the time required for assessment was reduced by 46.6% ± 25.8% across all cores, and by 57.8% ± 17% specifically for benign cores. These reductions were confirmed by Wilcoxon signed-rank tests (p *<* 0.05).

FIG. S1. **Visualization of the discriminative patches (A. - D.) from WSIs overlaid with attention maps from the TimeSFormer model determined to be tumor patches containing volumetric information important to detecting clinical significance.**

Although the coregistration step introduces a fixed computational delay of approximately 5 minutes per core, this is a one-time cost that is incurred prior to review. The significant downstream reduction in diagnostic time suggests that the method provides net efficiency gains, particularly in high-throughput or screening scenarios where manual serial review is especially time-consuming.

FIG. S2. **Time savings with 2.5D viewer across diagnostic categories.**

1. Note1. -@x https://github.com/facebookresearch/TimeSformer.
2. Note2. @x https://github.com/yiyixuxu/TimeSformer-rolled-attention.
